# Fabrication of TiO_2_/NiO p-n Nanocomposite for Enhancement Dye Photodegradation under Solar Radiation

**DOI:** 10.3390/nano12060989

**Published:** 2022-03-17

**Authors:** Mohamed Zayed, Salsbeel Samy, Mohamed Shaban, Abeer S. Altowyan, Hany Hamdy, Ashour M. Ahmed

**Affiliations:** 1 Nanophotonics and Applications (NPA) Lab, Physics Department, Faculty of Science, Beni-Suef University, Beni-Suef 62514, Egypt; m.zayed88ph@yahoo.com (M.Z.); salsabeelsamyabdelaziz@gmail.com (S.S.); hshamdy@hotmail.com (H.H.); ashour.elshemey@gmail.com (A.M.A.); 2 Department of Physics, Faculty of Science, Islamic University in Madinah, Al-Madinah, Al-Munawarah 42351, Saudi Arabia; 3 Department of Physics, College of Science, Princess Nourah bint Abdulrahman University, P.O. Box 84428, Riyadh 11671, Saudi Arabia

**Keywords:** TiO_2_/NiO nanocomposite, heterojunction, photocatalytic, dye degradation

## Abstract

A p-n nanocomposite based on TiO_2_ nanotubes (NTs) and NiO nanoparticles (NPs) was designed and optimized in this study to improve the photocatalytic performance of methylene blue (MB). The hydrothermal technique has been used to produce TiO_2_/NiO nanocomposites with different NiO NPs weight ratios; 1TiO_2_:1NiO, 1TiO_2_:2NiO, and 1TiO_2_:3NiO. The crystal phase, chemical composition, optical properties, and morphology of TiO_2_/NiO were explored by various techniques. TiO_2_ NTs have a monoclinic structure, while NiO NPs have a cubic structure, according to the structural study. The bandgap of TiO_2_ NTs was reduced from 3.54 eV to 2.69 eV after controlling the NiO NPs weight ratio. The TiO_2_/2NiO nanocomposite showed the best photodegradation efficiency. Within 45 min of solar light irradiation, the efficiency of MB dye degradation using TiO_2_/2NiO hits 99.5% versus 73% using pure TiO_2_ NTs. Furthermore, the catalytic photodegradation efficiency did not deteriorate significantly even after five reusability cycles, intimating the high stability of the TiO_2_/2NiO nanocomposite. This suggests that the loading of NiO NPs into TiO_2_ NTs lowers the recombination of photo-produced electron/hole pairs and enlarged solar spectral response range, which results in improved photocatalytic activity. The mechanism of charge transfer in the TiO_2_/NiO and kinetic models were discussed for the photodegradation of MB.

## 1. Introduction

Wastewater from the various industrial processes of printing and textiles contains significant amounts of organic compounds, which contribute to increased pollution of the environment. Indeed, about 10% of the world’s total dye supply is lost and released into the water during the dying process [1,2]. This is considered one of the most significant causes of water contamination. The existence of dye toxins in water supplies reduces the consistency of water, which can have harmful effects on humans and the surrounding wildlife [3]. The decolorization of toxic dye wastes has, therefore, gained more and more attention. Several methods have been applied as potential methods to remove dyes from water to prevent their side effects. These methods include adsorption, advanced oxidation, filtration, coagulation, flocculation, photocatalytic, and microbial degradation [4,5]. Among these techniques, photocatalytic degradation is recommended as a low-cost, environmental, and high-efficient method of oxidizing organic pollutants [6,7].

Several semiconductor photocatalysts, such as CuO, ZnO, TiO_2_, NiO, and Fe_2_O_3_, were used in developing photocatalysts for dye degradation [8,9,10,11,12]. Many studies have been focused on TiO_2_ as an n-type semiconductor for the treatment of many toxic pollutants [13,14,15]. This is due to TiO_2_ having highly desirable properties, such as low cost, heavy oxidization, non-toxic nature, and high photocorrosion stability [16]. Unfortunately, two main practical issues are accompanied with the usage of a TiO_2_ photocatalyst. First, the rapid recombination of photoelectron-hole pairs in TiO_2_ reduces photocatalytic performance [17]. Second, it has large bandgap energy [18]. This means it requires a photon with a wavelength in the ultraviolet (UV) region for electron-hole pair production. Less than 5% of solar energy is UV irradiation. Therefore, many more research investigations are needed to boost TiO_2_′s catalytic activity to meet the demands of practical applications.

Doping, deposition of noble metal, and coupled semiconductor are widely used to improve the photocatalytic properties of TiO_2_. More focus was given to the paired semiconductors, such as SnO_2_, CuO, ZnO, Fe_2_O_3_, and NiO nanoparticles, with TiO_2_ to build a heterostructure nanocomposite. This is because heterostructures can enhance the charge separation, suppress the electron-hole recombination rate, and tune the bandgap to the visible region [19,20,21]. The combination of coupled semiconductors with nanoparticles of a vast surface area can also lead to a greater adsorption capacity. There have been several TiO_2_-based heterostructure systems introduced and explored. Liu et al. [22] prepared TiO_2_/CuO heterojunction by spray drying process for photodegradation of methyl orange solution. The degradation percentage reaches only 65% after a time of 60 min under irradiation. Lv et al. [23] prepared TiO_2_/BiVO_4_ heterojunction for the degradation of Rhodamine B by a hydrothermal method. Full photodegradation is achieved for a long time after 4 h of exposure to visible light. Cao et al. [24] synthesized TiO_2_/Fe_2_O_3_ by using atomic layer deposition. The highest photodegradation activity of TiO_2_/Fe_2_O_3_ was 97.4% after 90 min. Nasr et al. [25] synthesized TiO_2_/BiOF heterostructures via a solid-state sintering method. There was a slight improvement in the visible light absorption. Furthermore, the fabrication method is done in the existence of hazardous hydrofluoric acid solution and argon atmosphere.

On the other side, nickel oxide (NiO) is an excellent cocatalyst for improving the photocatalytic performance of TiO_2_ for several reasons [26,27,28,29,30]. NiO is an important p-type semiconductor of wide bandgap energy (3.50 eV) [31]. It has excellent properties included as great carrier concentration, superior stability, and absorption light in the visible region. Furthermore, the unique electronic structure of NiO leads to the fast mobility of charge carriers. For a TiO_2_/NiO nanocomposite, the n-type TiO_2_ areas collect positive charges, while the p-type NiO areas collect negative charges [31]. Hence, charge equilibrium is established, resulting in improved interfacial charge separation and longer charge carrier lifetimes [32,33]. Consequently, TiO_2_/NiO heterostructures have gained more interest. The hydrothermal approach is widely utilized for the creation of novel materials and nanostructures because it has numerous advantages, including low-cost, simplicity, controllability, and high yield. It is also environmentally benign because the reactions are carried out in a closed system at low temperatures [34]. 

Although there are many previous works on the photodegradation activity for TiO_2_/NiO nanocomposite, the photocatalytic efficiency is still low and the time of irradiation is long. Furthermore, optimizing the ratio between TiO_2_ and NiO was not addressed. J. Liu et al. [35] prepared the TiO_2_/NiO by hydrothermal technique, but they achieved only 75% removal efficiency for MB after a 90 min reaction time under a 300 W Xenon lamp. M. Sabzehparvar et al. [36] reached a removal efficiency of 85.5% for MB after a 90 min irradiation time using TiO_2_/NiO/Ag nanocomposite. Y. Yang et al. [37] prepared a TiO_2_/NiO/Ni nanocomposite thin film via sol-gel method followed by calcination. The efficiency reached about 23.5%, 74.4%, and 98.0% for MO (methyl orange), RhB (rhodamine B), and MB, respectively, at a concentration of 2 mg/L under a solar simulator with a light intensity of 510 W/m^2^. N. J. Baygi et al. [38] fabricated TiO_2_/NiO by a modified combustion-based process. The degradation percentage of MB is less than 90% after 210 min under daylight emission. Furthermore, the stability was not investigated. M. Faisal et al. [39] prepared TiO_2_/NiO in the presence of triblock copolymer as a directing agent. Approximately 90% of the MB dye was gone after 180 min of visible light exposure. H. Rojas et al. [40] designed a TiO_2_-F/NiO nanocomposite by in situ fluorination of the semiconductor using a sol-gel method. The photocatalytic efficiency achieved 88% for caffeine degradation after 180 min under UV irradiation. Hence, enhancement of the photocatalytic performance of the TiO_2_/NiO for dye photodegradation within a short time is highly required.

In this study, TiO_2_/xNiO nanocomposites were synthesized using a simple and inexpensive hydrothermal technique and physical mixing solid-solid interaction. Furthermore, optimization of the weight ratio of NiO NPs content for p-n nanocomposite photocatalysts was presented. The structural properties, optical properties, and chemical composition of the fabricated nanopowders are identified by SEM, XRD, EDX, FT-IR, and UV/Vis spectrophotometry. The photocatalytic performances of the nanopowders were studied by using MB dye under sunlight and a 400 W Xenon lamp. The photodegradation process’s stability, mechanism, and kinetics are studied.

## 2. Experimental Details

### 2.1. Chemicals and Materials

Nickel chloride dihydrate (NiCl_2_·6H_2_O, Alfa Aesar, Kandel, Germany, 99%), hydrochloric acid (HCl, Fluka, Munich, Germany, 96%), urea (CO(NH_2_)_2_ LOBA Chemie, Mumbai, India, 98%), titanium dioxide (TiO_2_, Alfa Aesar, Kandel, Germany, 99%), and sodium hydroxide (NaOH, Fluka, Munich, Germany, 98%) were used as received from the supplier.

### 2.2. Synthesis of TiO_2_ NTs

Pure TiO_2_ nanostructures were fabricated by the hydrothermal technique with the calcination process. About 200 mM of TiO_2_ was added to a 10 M NaOH aqueous solution and magnetically stirred for 60 min. Then, the mixture was placed in a Teflon-lined stainless steel autoclave for hydrothermal treatment at 140 °C for 24 h in a muffle furnace. The autoclave was cooled to room temperature when the reaction was completed and the white product precipitate was separated. The precipitate was washed with 100 mM HCl acid solution and deionized (DI) water several times until almost all of the Na^+^ ions were removed [41]. Lastly, the precipitate was filtered, dried at 60 °C in an oven, and then annealed for 3 h at 400 °C. The nanopowder was recorded as TiO_2_ NTs.

### 2.3. Synthesis of NiO NPs

Equi-molar (0.2 M) nickel chloride and urea were dissolved in DI water at room temperature for 30 min under magnetic stirring. Then, the obtained solution was placed in the autoclave and hydrothermally treated for 16 h at 140 °C. After cooling to room temperature, the product was centrifuged and rinsed multiple times with distilled H_2_O. Finally, the nanopowder was calcined for 3 h at 400 °C for the complete conversion into NiO NPs crystalline phase.

### 2.4. Nanocomposite TiO_2_/NiO Loading

TiO_2_/NiO nanocomposite was prepared from a nanopowders mixture of the corresponding component solid oxides. Different weight ratio TiO_2_ NTs and NiO NPs mixture and ground thoroughly in an agate mortar. To obtain an aqueous dispersion, the mixed oxides were ultrasonically disseminated in 45 mL distilled H_2_O for 2 h. Then, the suspension solution was stirred for 4 h. After filtering, the resulting precipitation was collected and dried in an oven for 15 h at 70 °C. The nanopowders were recorded as TiO_2_/xNiO, and the x (=1, 2, and 3) was denoted to the adding weight of NiO NPs.

### 2.5. Characterization

The surface morphologies of TiO_2_ NTs, NiO NPs, and TiO_2_/xNiO nanopowders were observed by scanning electron microscopy (SEM; JEOL, JSM-5410LV, Tokyo, Japan). The degree of crystallization and phase composition were determined using X-ray diffraction (XRD; Philips X’Pert Pro MRD, Almelo, The Netherlands) with Cu-K radiation at 40 kV in the 2θ-range 20°–80° with scan rate 0.05°/s. The morphology and structure of samples were characterized utilizing transmission electron microscopy (TEM, JEOL JEM-2100) at 200 kV. The chemical compositions were examined by using the energy dispersive X-ray (EDX; JEOL JED-2300, Tokyo, Japan) spectrometer at 30 kV. The optical properties of the nanopowders were studied by using UV/Vis/IR double beam spectrophotometer (PerkinElmer, Lambda 950, Waltham, MA, USA). Fourier transform infrared (FTIR) charts of the designed nanopowders have been examined using Vertex 70, Mumbai, India, FTIR-FT Raman spectrometer.

### 2.6. Photocatalytic Activity Test

The photocatalytic properties of TiO_2_ NTs, NiO NPs, and TiO_2_/xNiO were evaluated by adopting MB as a model pollutant under direct solar light irradiation for time intervals up to 50 min. The experiments are carried out at pH 6. The photodegradation of MB was performed in a 200 mL beaker with a working volume of 100 mL MB solution. For residual MB concentration, the dye solution was collected and examined at a regular time interval by using a UV/Vis/IR spectrophotometer (PerkinElmer Lamba 950, Waltham, MA, USA) at a single wavelength of 664 nm (characteristic absorption wavelength of MB dye). All measurements were carried out in triplicate and the average values were reported. The photocatalytic experiments were carried out in February 2021 at Beni-Suef city (Egypt) on bright sunny days under clear sky conditions from 8 a.m. to 4 p.m. with a temperature of 25 °C. To reduce the effect of solar radiation variance, the trials were conducted on consecutive days and at a set hour. For comparison, the dye degradation was examined under a 400 W Xenon lamp (Jiangsu, China). The photodegradation performances have been tested after the adsorption/desorption equilibrium. The measurements were measured as a function of illumination time, MB initial concentration, and photocatalyst dose. Furthermore, the photocatalyst stability was studied for several photodegradation runs at different exposure times.

#### 2.6.1. Influence of Illumination Times and MB Initial Concentrations

The influence of illumination times and MB initial concentrations on the catalytic performance of the catalyst was tested. Under sun light, 0.05 g of TiO_2_/xNiO nanopowders were mixed with 100 mL MB dye solutions of various concentrations: 5, 10, 30, 50, 70, and 90 mg/L.

#### 2.6.2. Influence of the TiO_2_/NiO Catalyst Dose

The influences of the photocatalyst dose on the photocatalytic activity were studied using various TiO_2_/2NiO doses; 0.015, 0.020, 0.025, 0.050, and 0.100 g. The measurements were carried out using 100 mL of MB solutions of concentration 5 mg/L.

#### 2.6.3. Stability and Reusability of TiO_2_/NiO Catalyst

The TiO_2_/2NiO reusability was tested for five runs under sunlight using 0.05 g of the photocatalyst and 100 mL MB solution of concentration 5 mg/L. The photocatalyst nanopowder was rinsed with distilled H_2_O and ethanol and dried for 1 h at 60 °C after each cycle before being re-dispersed into a fresh MB solution for the following cycle.

## 3. Results and Discussion

### 3.1. Characterizations of the Fabricated Nanocomposite

#### 3.1.1. Structure Analysis

**Figure 1:**
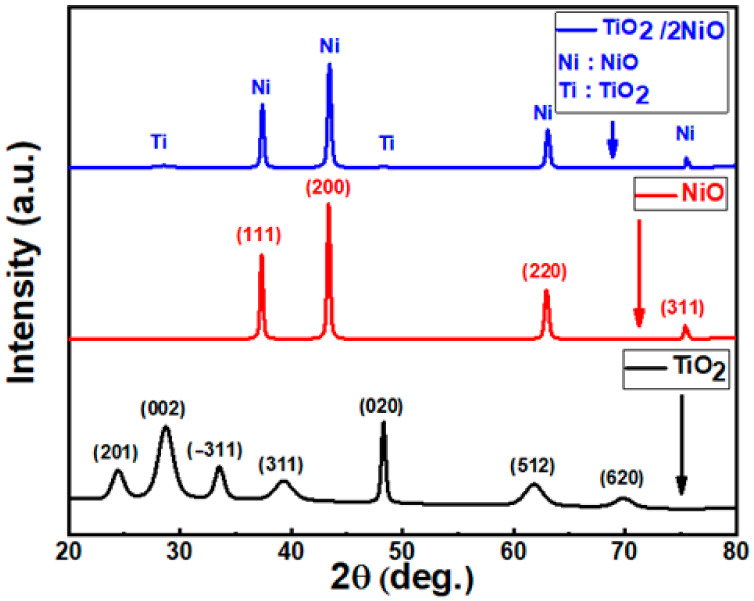
XRD charts of the fabricated TiO_2_ NTs, NiO NPs, and TiO_2_/2NiO.

XRD charts for the pure TiO_2_ NTs, NiO NPs, and TiO_2_/2NiO are displayed in Figure 1. Pure TiO_2_ NTs is a monoclinic structure based on JCPDS No. 04-009-8327 [42]. It has seven diffraction peaks at 2θ = 24.4°, 28.69°, 33.51°, 39.28°, 48.24°, 61.76°, and 69.81° as seen in Figure 1. These diffraction peaks were attributed to (201), (002), (−311), (311), (020), (512), and (620) planes, respectively. Multiple orientations were detected in the TiO_2_ NTs signifying that the TiO_2_ NTs are polycrystalline. These peaks also have high widths that suggest a tiny crystallite size. For the pure NiO NPs, there are four diffraction peaks located at 37.29°, 43.32°, 62.9°, and 75.4°, based on JCPDS No. 00-047-1049 [43]. These diffraction angles were corresponding to (111), (200), (220), and (311) planes, respectively, with cubic structure. The peaks of NiO NPs are narrow with high intensities peaks, proposing that the nanopowder is well crystallized. The TiO_2_/2NiO nanocomposite has well-indexed peaks related to the combination of cubic NiO NPs (JCPDS 00-047-1049) and monoclinic TiO_2_ NTs (JCPDS 04-009-8327). No characteristic peaks are detected for any impurities. Compared with TiO_2_ NTs, the TiO_2_/2NiO presented more sharp diffraction peaks and higher crystallinity. The intensity of diffraction peaks associated with TiO_2_ NTs is lower than that obtained for the TiO_2_/2NiO nanocomposite. After NiO NPs are loaded, the phase of TiO_2_ NTs does not change.

The crystallite size of nanopowders is obtained from the broadening of diffraction peaks by using the Scherer formula (1) [44].

(1) $$D=\frac{0.94\;\lambda }{\;\beta \;\cos\theta }$$

Here λ is the Cu-Kα X-ray wavelength (0.154 nm), β is the peak full width at half maximum (FWHM), and θ is the XRD angle at the center of the peak. The average crystallite size for TiO_2_ is 12.6 nm, with the largest crystallite size of 25.3 nm reported for the (020) orientation. The average crystallite size for NiO is 35.5 nm, while the (200) crystallite size is 35.9 nm. The average crystallite size for TiO_2_/2NiO is 35.6 nm, with a (200) crystallite size of 31.3 nm.

The interplanar distance between crystal planes measured using Bragg’s equation [45]:

(2) $$d=\frac{\lambda }{2\;\sin\theta }\;$$

There’s a little change in the interplanar distance (d) of TiO_2_/2NiO nanocomposite compared with pure TiO_2_ NTs for the planes (002) and (020) as a result of a very small shift of peak position.

The preferred growth orientations are recognized if the texture coefficient (TC) is greater than one. The TC of the nanopowders can be determined from the following Equation (3) [46]:

(3) $$\mathrm{TC}\;\left(\mathrm{hkl}\right)=\frac{I\left(\mathrm{hkl}\right)\;/{\;I}_{0}\left(\mathrm{hkl}\right)}{N^{-1}\;\sum_{n}I\left(\mathrm{hkl}\right)\;/{\;I}_{0}\left(\mathrm{hkl}\right)}$$

Here I(hkl) is the observed peak intensity, I_o_(hkl) is the JCDPS standard peak intensity, and N is the total number of diffraction peaks. The preferred crystallite growth orientations have TC > 1. According to the TCs calculations, the preferred orientation for the formation of TiO_2_ nanocrystallites is (020) with TC = 2.22, but the preferred orientation for NiO crystallites is (200) with TC = 1.89. The TiO_2_/2NiO TC values for the highest four intense peaks, which correspond to NiO crystallites, were determined and given in Table 1. The highest TC value (1.86) was observed for the (200) orientation.

#### 3.1.2. Surface Morphological Analysis

**Figure 2:**
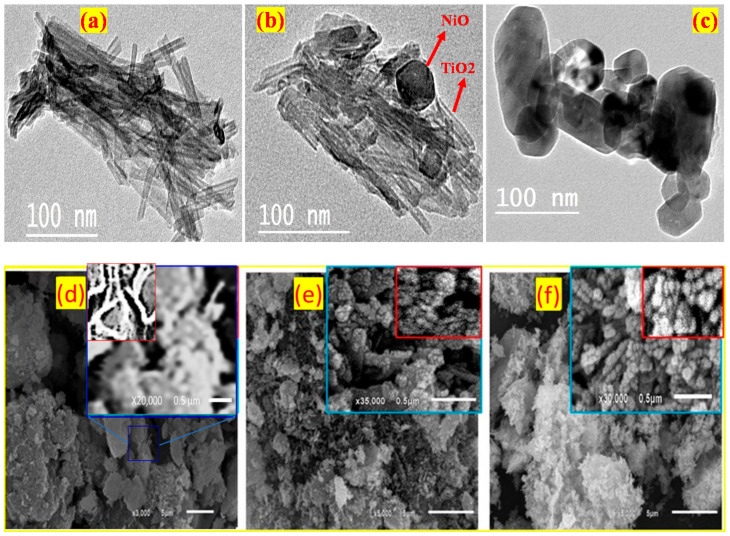
TEM images of the fabricated (**a**) TiO_2_ NTs, (**b**) TiO_2_/2NiO, and (**c**) NiO NPs, and SEM images of the fabricated (**d**) TiO_2_ NTs, (**e**) TiO_2_/2NiO, and (**f**) NiO NPs.

The TEM images of the TiO_2_ NTs, TiO_2_/2NiO, and NiO NPs samples are presented in Figure 2a–c. The TiO_2_ nanopowder, in Figure 2a, displays nanotubes with an average inner diameter of 4.4 ± 0.8 nm and an average outer diameter of 10.5 ± 0.9 nm. The wall thickness of TiO_2_ NTs is ~3.1 nm. Figure 2b shows the TEM image of the TiO_2_/2NiO sample. Mixed TiO_2_ nanotubes and NiO hexagonal nanoparticles are observed in this figure. The average diameter of NiO is 47.2 ± 1.5 nm, whereas the average inner and outer diameters of TiO_2_ are 5 and 10.9 nm, respectively. The TEM image of the NiO sample is composed of mixed morphologies from hexagonal nanorods and nanoparticles as seen in Figure 2c. The diameters of the NiO nanoparticles are ranged from ~33 to ~39 nm. These values are very close to the crystallite size obtained by the XRD analysis. The length of the nanorods is ranged from ~97 to 132 nm, whereas the width is ranged from ~44 to ~75 nm. It is important to mention that the TiO_2_ nanotubes can offer higher photo activities compared to other forms of TiO_2_, such as nanorods (NRs), nanowires (NWs), and nanoparticles (NPs). This is due to their hollow structure, which increases the surface area and light absorption [34,47]. Furthermore, the nanotubes work as channels for facilitating the electron and ion transport at the interfaces of the photocatalyst/dye. Because of the significant charge transfer, the development of a p-n heterojunction at the interface of TiO_2_ NTs and NiO NPs can greatly boost photocatalytic activity and delay electron-hole recombination [48,49,50].

Furthermore, the morphologies of the photocatalysts were examined using the SEM images in Figure 2d–f. Figure 2d indicates that the fabricated TiO_2_ are nano/micro agglomerates of nanotubes with rough surfaces. This rough surface was favorable to the loading of NiO NPs. The magnified SEM images in Figure 2d show that the surface of the agglomerates is made of densely packed agglomerated nanotubes, as shown in the inset SEM image. Figure 2e shows the surface morphology of the TiO_2_/2NiO nanocomposite. The TiO_2_/2NiO nanocomposite is composed of TiO_2_ micro/nanoparticles partially covered with fine and homogeneous NiO NPs, as seen in the high-magnification image. Therefore, when the TiO_2_ NTs surface was loaded with NiO NPs that could be linked to the protrusions, the grains were not visible. This suggests to the formation of the TiO_2_/2NiO p-n heterojunction. The NiO NPs possess an aggregated nano/microsphere-like morphology, as shown in Figure 2f. The microsphere contains small nanoparticles with a regular arrangement. Figure S1(a,b) (supplementary data) shows histograms for the particle size distribution of the NiO and TiO_2_/2NiO nanoparticles obtained from the inset SEM images of Figure 2e,f. The average particle size is ~40 nm for NiO and ~70 nm for TiO_2_/2NiO.

#### 3.1.3. EDX Study

**Figure 3:**
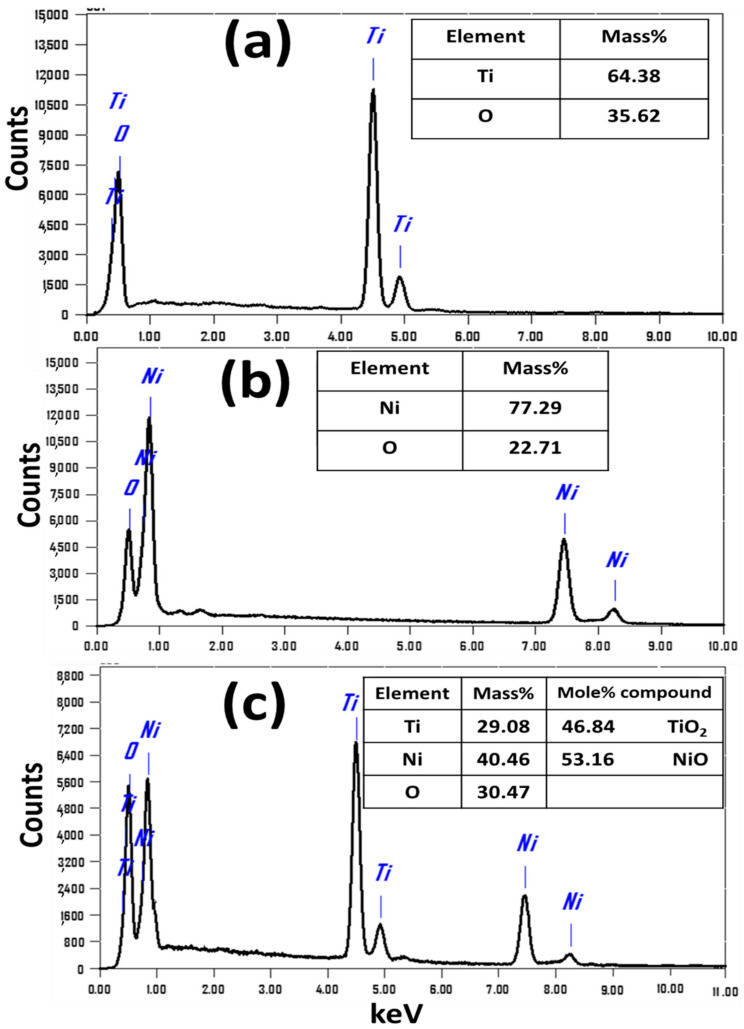
EDX spectra for (**a**) TiO_2_ NTs, (**b**) TiO_2_/2NiO, and (**c**) NiO NPs. The inset Tables show the quantitative EDX analysis.

The prepared nanopowders were examined by EDX to identify quantitative and qualitative chemical composition. Figure 3 shows the EDX patterns of (a) TiO_2_ NTs, (b) NiO, and (c) TiO_2_/2NiO NPs at the microscopic level with inset tables providing quantitative analysis. For TiO_2_ NTs, there are only signals for Ti and O with mass ratios of 64.38% and 35.62%, respectively. No impurities of elements, such as Na and C, were found within the detection limit of the EDX. This indicates that the high purity and well-crystallization of the fabricated TiO_2_ NTs agree well with the XRD results. The peaks Ti is positioned around are 4.9, 5.1, 4.5, and 4.8 keV. Furthermore, the obtained ratios are very close to the ideal stoichiometric ratio between Ti and O atoms. A typical EDX pattern of the fabricated NiO NPs is presented in Figure 3b. This EDX spectrum reveals the presence of Ni and O signals. According to EDX analysis, the mass ratios are 77.29% Ni and 22.71% O, which is quite close to the stoichiometric mass ratios of NiO (78.6% Ni, 21.4% O). The quantitative EDX analysis, in Figure 3c, shows that the TiO_2_/2NiO nanocomposite is composed of Ti, Ni, and O. The mole ratios for the compound TiO_2_ NTs and NiO NPs are 46.84 and 53.16%, respectively, which further confirm the successful formation of a TiO_2_/2NiO nanocomposite.

#### 3.1.4. Optical Characterization

**Figure 4:**
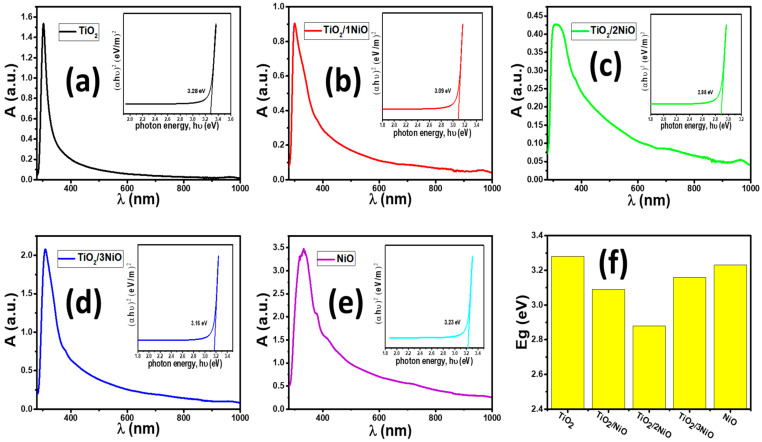
Absorbance spectra with inset figure of Tauc’s plot of (α hν)^2^ versus hν of (**a**) TiO_2_ NTs, (**b**) TiO_2_/NiO, (**c**) TiO_2_/2NiO, (**d**) TiO_2_/3NiO, and (**e**) NiO NPs; and (**f**) energy gap of all nanopowders.

The optical properties of a photocatalyst influence its photocatalytic activity. For enhancing the photocatalytic performance of titania, the absorption band and optical bandgap must be shifted towards the visible region for efficient solar light photons harvesting [31]. The TiO_2_/NiO heterostructure can display a good visible light response [51]. Consequently, optimizing the optical property of loading NiO NPs is highly desirable. UV/Vis/IR spectrophotometry is used to identify the optical properties and bandgap of the photocatalysts. The absorbance (A) spectra of the prepared photocatalysts are studied from 250 to 1000 nm and are represented in Figure 4a–e.

From Figure 4a, TiO_2_ NTs exhibit a strong absorption band with an edge in the UV region at around 305 nm corresponding to electron jump from O 2p valence band to Ti 3d conduction band [52]. This is in line with TiO_2_ NTs’ intrinsic band-gap absorption. The absorption spectrum decreases rapidly as the wavelengths increase from 300 to 1000 nm, so that the TiO_2_ NTs showed no significant spectral response to the visible light region. The sharp absorption edge confirms the good crystalline quality nature of the TiO_2_ NTs and the narrow size distribution of the nanoparticles [53,54]. This confirms the XRD and SEM measurements.

The prepared NiO NPs have a black color as a consequence of the lattice defects and their nanoscale size [55]. The absorption spectrum of the black NiO NPs exhibits a high response to the light, owing to a wavelength of nearly 700 nm. This suggests that NiO NPs exhibits visible light absorption response, decreased band gaps, and probably improved carrier collection, which significantly benefits the photocatalytic activity [32]. After loading NiO NPs on the TiO_2_ NTs, the color of TiO_2_ NTs, TiO_2_/NiO, TiO_2_/2NiO, and TiO_2_/3NiO powders change from white, to yellow to light grey, and, further, to dark grey due to the different loadings of NiO NPs. The right absorption edge of these nanopowders exhibits a redshift towards a higher wavelength at the visible region compared with that of pure TiO_2_ NTs. This shift is caused by the presence of more opportunities both for band overlapping and strong interaction between the TiO_2_ NTs and NiO NPs, which modifies the electronic structure of TiO_2_/NiO nanocomposite [56]. Furthermore, as a result of the formation of the p-n junction, good inter-dispersion of TiO_2_ NTs and NiO NPs semiconductors and contacts between them leads to an extended absorption edge in the visible region [30]. The interaction between Ti and Ni species leads to a reduction in the bandgap between Ti 3d and O 2p of TiO_2_ states and redshift in the absorption spectrum, which makes it easier to absorb the visible light [35,57,58]. As the NiO NPs content gradually increased, the absorption edge red-shifts gradually and the TiO_2_/2NiO had the maximum redshift. The absorption edge is blue-shifted with a further increase in the NiO NPs content. This behavior agrees with the previous works [59,60]. The blueshift at a high ratio of NiO in the TiO_2_/3NiO nanocomposite might be related to particle size owing to the quantum confinement effect. Furthermore, this trend can be explained by the synergetic effects that related to the electronic contact between both oxide particles. Consequently, the blueshift of the absorption edge would preclude visible light absorption for TiO_2_ NTs and limit the effect of the p-n heterostructure because of the excessive NiO NPs loading in the TiO_2_/3NiO nanopowder [61].

Hence, the TiO_2_/2NiO nanopowder has a great absorption band in the UV/Vis compared with the other nanopowders. This implies that the TiO_2_/2NiO nanopowder can respond to photons with visible light wavelengths (the maximum portion of solar light), which is better for charge transfer. Using the obtained data for absorbance (A), the absorption coefficient (α) was calculated by using the following equation [62]:

(4) $$\alpha \;=2.303\;\frac{A\;\rho }{L\;C}$$

where ρ is the photocatalyst density, L is the quartz cell width (1.0 cm), and C is the photocatalyst concentration in the suspension. The direct band gap of nanopowders was calculated by using the Tauc equation [54]:

(5) $${\left(\alpha \;h\nu \right)}^{2}=\;B\;\left(h\nu \;-\;\mathrm{Eg}\right)$$

Here B is a constant, ν is the frequency of the photon, h is Planck’s constant, Eg is the direct bandgap, and α is the absorption coefficient. From the plots of ${\left(\alpha \;h\nu \right)}^{2}$ versus hν and intersection of the linear portion with the hν axis, Eg= hν when α=0, as shown in the insets of Figure 4a–e. The obtained values of Eg are plotted in Figure 4f for all of the nanopowders. The value of Eg for the nanostructured TiO_2_ NTs is decreased by the incorporation of the NiO NPs for the nanopowders TiO_2_/NiO and TiO_2_/2NiO, which matches the observed redshift in the absorption edge. The bandgap of TiO_2_/3NiO nanopowder is then increased as the weight ratio of NiO NPs is raised.

The estimated values of the energy gaps of the TiO_2_ NTs, TiO_2_/NiO, TiO_2_/2NiO, TiO_2_/3NiO, and NiO NPs are 3.28, 3.09, 2.88, 3.16, and 3.23 eV, respectively, as clarified in the insets of Figure 4. The Eg values for TiO_2_ and NiO are matched well with the values reported in the previous works [32,37,63,64,65,66]. The energy gap between the Ti d and O p orbitals of TiO_2_ NTs may be reduced by the overlap of the Ti d orbital of TiO_2_ NTs and the Ni d orbital of NiO NPs at the interface area [65]. For nanomaterials with a narrower bandgap, electrons can more easily move from the V.B to the C.B. Based on the band gap values, the TiO_2_/2NiO nanocomposite’s optical absorption is clearly improved. Hence, TiO_2_/2NiO nanopowder is the most suitable for application in the photodegradation of dye.

#### 3.1.5. FT-IR Analysis

**Figure 5:**
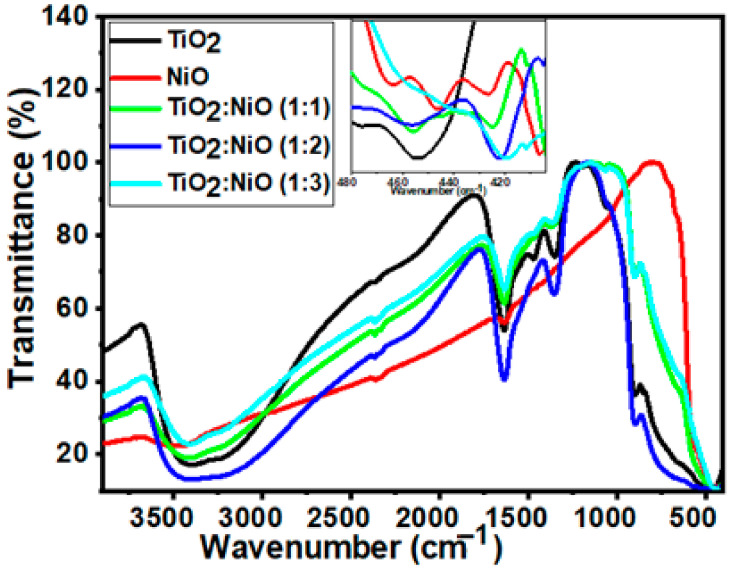
FTIR spectra of TiO_2_ NTs, NiO NPs, and TiO_2_/xNiO nanocomposite.

The presence of a functional group on the photocatalyst surface can be determined via infrared spectroscopy. The FTIR spectra of the samples were analyzed in the wavenumber range from 4000 to 400 cm^−1^, as displayed in Figure 5. Several broad absorption bands appeared indicating the nanocrystalline nature of the samples. The broadband that appeared between 3500 to 3000 cm^−1^ was ascribed to stretching hydroxyl group (O-H) vibration [67].

Two bands located between 2366 and 2338 cm^−1^ for pure TiO_2_ NTs are assigned to C-H stretching vibrations. These bands are shifted to 2365 and 2342 cm^−1^ for TiO_2_/3NiO, respectively. The peak at ~1635 cm^−1^ refers to the bending modes of Ti-OH [68]. This is evidence of a large number of water adsorbed molecules on the TiO_2_ NTs surface that play an important role in photodegradation performance [69]. The adsorbed OH ions trapped the charge carriers to produce reactive OH radicals, which act as active sites for degrading the dye molecules.

The prominent band located around 1360 cm^−1^ is related to Ti-O modes [70]. The band at 1060 cm^−1^ could be due to the Ti-O vibration [71]. Furthermore, the Ti-O vibration is observed at 895 cm^−1^. Moreover, the weak band around 455 cm^−1^ is related to the vibrational mode of the Ti-O-Ti bond in the anatase phase [72]. The band around 767 cm^−1^ is correlated to the band C=O stretching vibrations, which indicated that the NiO NPs powers tend to strong physical absorption of CO_2_ [73]. As seen in the inset of Figure 5, the Ni-O stretching vibration occurs at 470–400 cm^−1^ [74]. These peaks appear in the TiO_2_ and NiO nanocomposite, which suggests the interaction between NiO NPs and the TiO_2_ NTs blend matrix.

### 3.2. Dye Removal Study

#### 3.2.1. Influence of Illumination Time

**Figure 6:**
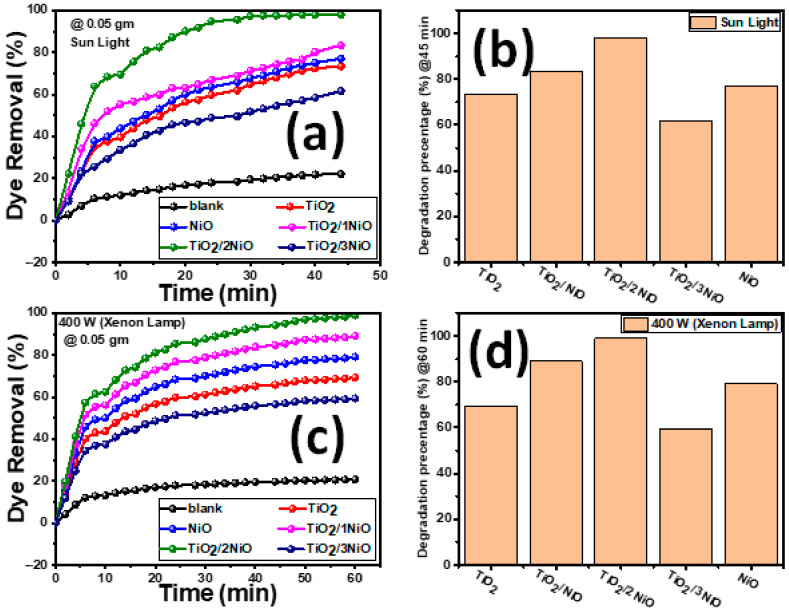
(**a**) The influence of sunlight illumination on the photodegradation of MB dye (5 mg/L) for all prepared nanopowder (0.05 g); (**b**) the degradation percentage of MB under sunlight irradiation for 45 min; (**c**) the effect of Xenon Lamp (400 W) irradiation on the photodegradation of MB dye (5 mg/L) for all fabricated nanopowder (0.05 g); and (**d**) the degradation percentage of MB under 400 W Xenon Lamp irradiation for 60 min.

The photocatalytic activities of the fabricated TiO_2_ NTs, NiO NPs, and TiO_2_/xNiO composites were tested for the photodegradation of MB dye under natural sunlight and a 400 W Xenon lamp. Figure 6 demonstrates the photocatalytic removal% versus the illumination time at an initial dye concentration of 5 mg/L and catalyst mass of 0.05 g. 

The photodegradation percentage $\eta \;\left(\%\right)$ of MB dye is determined based on the following Equation (6) [2].

(6) $$\eta \;\left(\%\right)=\left(\frac{C_{o}-C_{t}}{C_{o}}\right)\times 100$$

where $C_{o}$ the starting absorption of the MB solution and $C_{t}$ the absorption of MB dye after exposure time t. The overall behaviors for catalytic photodegradation of the examined MB dye by all nanopowders demonstrate a continual increase in dye removal% as illumination time increases. It could be seen in Figure 6a that the NiO NPs Wt% ratio exhibits a significant influence on the photocatalytic activity process compared with the pure TiO_2_ NTs.

The photocatalytic dye removal curves for MB suggest that pure TiO_2_ NTs have low photocatalytic activity because of their high bandgap [75,76]. Furthermore, NiO NPs have rapid recombination of photogenerated electrons-holes [77,78,79,80]. Therefore, only a fraction of the electrons and holes were involved in the photocatalytic response, leading to low photocatalytic activity. The coupled heterostructures between TiO_2_ NTs and NiO NPs exhibited superior photocatalytic behavior. The decomposition of MB in the absence of the catalyst under sunlight and 400 W Xenon lamp illumination is presented in Figure 6a,c (black color). The dye degradation is about 20% after 45 min under sunlight illumination and after 60 min under 400 W xenon lamp illumination. This suggests that the MB self-decomposition is limited in the absence of the catalyst. From Figure 6a,c, the removal efficiency is higher under sunlight irradiation than under Xenon lamp irradiation at the same exposure time. From Figure 6b,d, the values of the photodegradation% of MB dye using the photocatalysts TiO_2_ NTs, TiO_2_/1NiO, TiO_2_/2NiO, TiO_2_/3NiO, and NiO NPs after 45 min under sunlight illumination are 73.4, 83.3, 99.2, 61.5, and 77%, respectively. Furthermore, the percentage photodegradation values of MB dye using the photocatalysts TiO_2_ NTs, TiO_2_/1NiO, TiO_2_/2NiO, TiO_2_/3NiO, and NiO NPs after 60 min under 400 W Xenon lamp illumination are 69.28, 89.07, 98.97, 59.39, and 79.18%, respectively. 

This behavior matches with optical properties (Figure 4). For MB dye concentration of 5 mg/L, the TiO_2_/2NiO nanopowder exhibits the best photocatalytic activity, which reached complete photocatalytic degradation (~100%) after 30 min and then reaches a plateau value, as seen from Figure 6a. The improved photocatalytic performance of TiO_2_/2NiO nanopowder is ascribed to a collective effect of many factors: First, the TiO_2_/2NiO nanopowder shows the highest surface-volume ratio, which increases the light absorption rate, provides more reactive sites for adsorption of MB, and gives a positive influence towards the high photocatalytic activity. Second, TiO_2_/2NiO nanopowder has higher separation efficacy for the electron/hole pairs and prolongs the separation lifetime, which reacts with H_2_O and O_2_ further to degrade MB. Besides, the TiO_2_/2NiO nanopowder has narrow Eg, which provides more electron-hole pairs under visible light region. The efficient creation of essential oxidizing species, such as ${\mathrm{OH}}^{.}$ and $O_{2}^{.-}$ radicals, which are the major factor for the destruction of the targeted pollutants, derive from the rapid flow of electrons and holes to the surfaces of composites [39]. By increasing NiO NPs content in the nanocomposite, the photocatalytic activity rapidly decreases, as noticed in the TiO_2_/3NiO nanopowder. The same behavior was observed in many previous works [81]. This was possibly due to the accumulation of excessive NiO clusters, which shield the active sites on the TiO_2_ NTs surface from touching the dye molecules and decreased the light absorption of TiO_2_ NTs. At the same time, the rapid recombination rate of the electron/hole pairs in NiO NPs reduces the quantum efficiency of the TiO_2_/3NiO. Besides, because of the high electron affinity of TiO_2_ NTs relative to NiO NPs, the lifetime of the electron-hole pair decreases. Furthermore, the recombination of the accumulated holes with the photogenerated electrons reduces the photocatalytic activity [16]. From the above results, the TiO_2_/2NiO nanopowder indicates a very high efficiency in the MB photodegradation under sunlight in a short period compared to other nanopowders. Within a short time using TiO_2_/2NiO, the degradation efficiency of MB under sunlight was sharply increased relative to the efficiency of the pure TiO_2_ NTs. Accordingly, the TiO_2_/2NiO nanopowder can be considered as the optimized nanopowder and will be used for subsequent works.

#### 3.2.2. Kinetic Modeling

To obtain a better comparison of the photocatalytic efficiencies for prepared nanopowders, kinetic models’ analysis of the TiO_2_/xNiO for catalytic photodegradation of MB was performed under sunlight illumination. The kinetic models explored are the zero-order, first-order, and second-order models, which can be stated by Equations (7)–(9), respectively [82]:

(7) $$C_{t}={\;C}_{0}-{\;k}_{0}\;t$$

(8) $$C_{t}={\;C}_{0}{\;e}^{-k_{1}\;t}$$

(9) $$1/C_{t}=1/C_{0}+{\;k}_{2}\;t$$

here, k_n_ indicates the photodegradation rate constant (n = 0, 1, 2). C_0_ is the starting MB concentration and C_t_ is the reminder MB concentration after exposure time t. 

**Figure 7:**
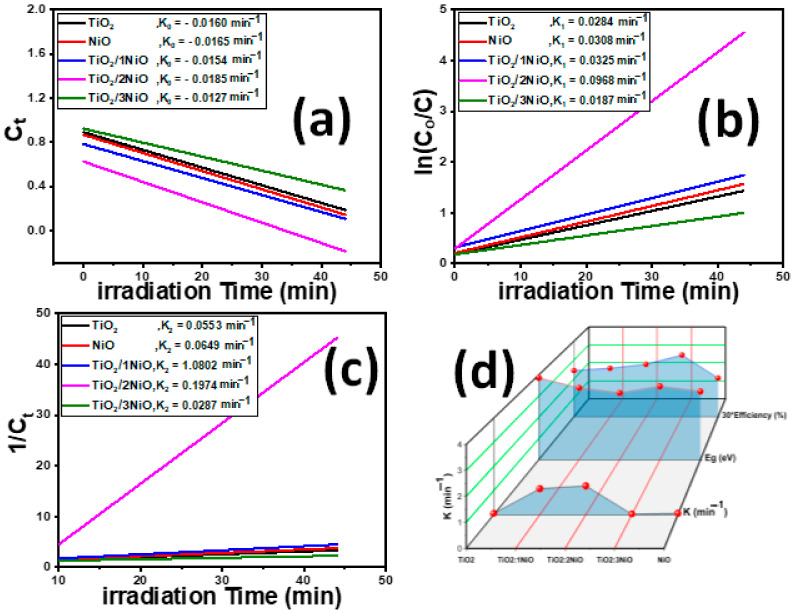
Kinetic study of the catalytic photodegradation of MB solutions using the prepared nanopowders under sunlight (**a**) zero-order, (**b**) first-order, and (**c**) second-order kinetic models, and (**d**) kinetic constants vs. Eg and Efficiency.

Fittings of the catalytic photodegradation of MB dye using TiO_2_/xNiO nanocomposite with the zero-order, first-order, and second-order models were evaluated through linear regression plotting of C_t_, ln(C_0_/C_t_), and 1/C_t_ vs. time, respectively, as illustrated in Figure 7a–c. The slopes of the straight lines represent the catalytic photoreaction rate constants (k). The obtained values of the photodegradation rate constants and correlation coefficients (R^2^) for all TiO_2_/xNiO nanopowders at starting MB concentration of 5 mg/L are listed in Table 2. The TiO_2_/2NiO nanopowder has a high kinetic degradation rate constant for all models.

Fitting of the zero-order kinetic model indicated that the TiO_2_/2NiO nanopowder has a high value of the kinetic rate constant (k_0_ = 0.01848 min^−1^) and very poor fitting (R^2^ = 0.67713) compared with other nanopowders (Table 2). The degradation rate constant of the first-order model gradually increases with increasing the weight ratio of NiO NPs in the TiO_2_/NiO nanocomposite from 1:1 to 1:2, and then it is decreasing for TiO_2_/3NiO and pure NiO NPs. The fitting value of the degradation rate constant for TiO_2_/2NiO nanopowder is 1.19736 with a good correlation coefficient (R^2^ = 0.90219). The data of the second-order kinetic model indicate the medium fitting of the obtained data for TiO_2_/NiO and TiO_2_/3NiO, while the high fitting for the degradation of TiO_2_/2NiO. Furthermore, the degradation rate constant for TiO_2_/3NiO exhibits a high value (k_2_ = 0.09682) compared to other nanopowders. From the above analysis, the photodegradation of MB dye at starting concentration of 5 mg/L is better represented by the second-order model than by the others. 

#### 3.2.3. Influence of the MB Concentrations

**Figure 8:**
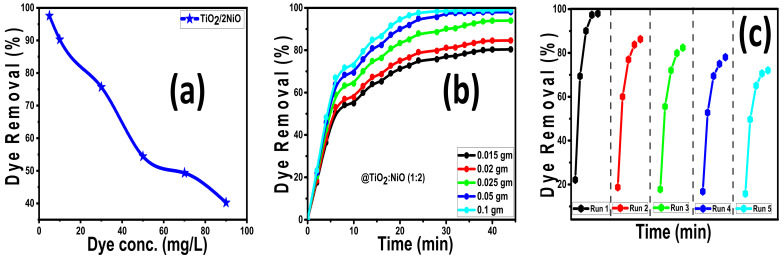
Under sunlight (**a**) the effect of the initial dye concentration on the photodegradation for TiO_2_/2NiO nanopowder, (**b**) the influence of the photocatalyst dose on photodegradation for TiO_2_/2NiO nanopowder, and (**c**) the reusability of TiO_2_/2NiO nanocomposite for the photodegradation of MB for five cycles.

The influence of MB dye concentration (5, 10, 30, 50, 70, and 90 mg/L) on photocatalytic removal% was studied for TiO_2_/2NiO nanopowder and presented in Figure 8a. In general, the catalytic photodegradation curves refer to a decrease in the removal% with increasing the dye concentrations at the selected irradiation time (45 min). The best photocatalytic results for TiO_2_/2NiO at 5 mg/L dye concentration were nearly 100% and then decreased to 42.5% for 90 mg/L MB dye concentration, as in Figure 8a.

The rise in the quantity of adsorbed dyes on the photocatalyst surfaces causes this behavior. Furthermore, the excessive dye concentration blocks incident light, preventing the necessary light intensity from reaching the photocatalyst. This reduces the number of hydroxyl radicals and positive holes produced, lowering the percentage of degradation [83]. As a consequence of loading NiO NPs onto TiO_2_ NTs at a weight ratio of 1:2, the resulting material is a fantastic photocatalytic nanomaterial for the elimination of high MB concentrations in a short time using sunlight illumination.

#### 3.2.4. Influence of the Catalyst Mass

The influence of the TiO_2_/2NiO catalyst mass (0.015, 0.02, 0.025, 0.05, and 0.1 g) on the photocatalytic removal of MB dye (5 mg/L) at 45 irradiation time was presented in Figure 8b. At a starting MB concentration of 5 mg/L, the proportion of dye removed increases as the catalyst mass increases. About 83.39%, 90.06%, and 94.56% for the 5 mg/L MB dye are removed after only 20 min sunlight exposure time, utilizing 0.025, 0.05, and 0.10 g of TiO_2_/2NiO nanocomposite, respectively. By increasing the exposure time to 30 min, the photodegration% increased to 96.7% and 99%, utilizing 0.05 and 0.10 g, respectively. This could be due to an increase in photogenerated hydroxyl radicals and positive holes when the photocatalyst dose is increased, as well as an increase in adsorption capacity when the total surface area is increased [84].

#### 3.2.5. Reusability of the Catalyst

The most significant elements for the practical utility of a photocatalyst during reactions are its reusability and stability. For five dye degradation cycles, the stability of TiO_2_/2NiO photocatalyst was investigated. This research involved treating 100 mL of MB solution of concentration 5 mg/L with 0.05 g of the photocatalyst for up to 45 min under sunlight illumination. After each run, the loss in photocatalyst dose due to the washing process was determined, and the error was included in the removal% estimate. From Figure 8c, the photocatalytic removal of MB dye decreased from 100 to 72.63% after five runs, suggesting good stability of TiO_2_/2NiO. The degradation curves of the investigated runs exhibit no equilibrium stage, indicating that the catalyst has the potential to remove more MB dye by extending the solar irradiation period. As a result, TiO_2_/2NiO might be a promising photocatalyst for large-scale environmental cleaning, because it may be easily isolated from the slurry system after the photocatalytic process using filtering. As a result, they can be reused more easily than traditional photocatalytic nanomaterials. For comparison, Table 3 shows the obtained results with various types of photocatalysts of previous works.

#### 3.2.6. Photocatalytic Mechanism of TiO_2_/NiO

**Figure 9:**
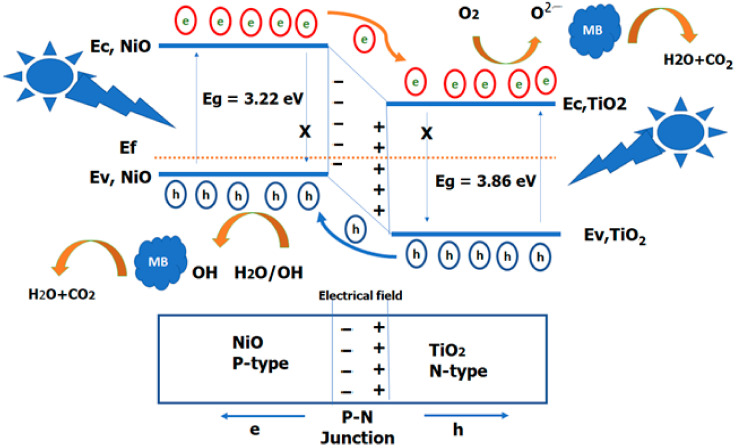
Schematic diagram of the charge transfer in TiO_2_/NiO-based photocatalyst.

The mechanism of photocatalysis is dependent on the generation of electrons and holes under light excitation, as presented in Figure 9. The catalytic photodegradation% is controlled by the amount of absorbed light, which validates the number of e-h pairs, and the separation of the charge carriers. Electrons may transfer to the conduction band in pure TiO_2_ NTs, leading to the creation of holes on the valence band (O 2p → Ti 3d) when TiO_2_ NTs is illuminated by UV light [90]. Photo-generated electrons and holes recombine to achieve a lower energy level.

The majority charge carrier production and recombination are two competing processes that influence catalytic photodegradation efficiency. Recombination of electrons/holes reduces carrier mobility and stops the majority of charge carriers from participating in the reactions. Before recombination, electrons and holes can react with harmful molecules and break them down into non-toxic compounds if they migrate to the surface of the semiconductor photocatalyst. For a TiO_2_/NiO nanocomposite, the formation of a p-n nanocomposite is an effective approach to increase the yield of photocatalytic reactions. To explore the effect of p-n type junctions on the performance of the nanocomposite, Figure S2 (supplementary data) shows the photocatalytic performance of TiO_2_/2NiO composite versus the performance of TiO_2_/2NiO mixture. After 60 min, the degradation efficiency using a TiO_2_/2NiO composite reached 99.8%, and using a TiO_2_/2NiO mixture reached 58.9%.

A schematic energy band diagram of the n-TiO_2_/p-NiO heterojunction structure is plotted in Figure 9. For n-type TiO_2_ NTs, the Fermi energy level is closer to the conduction band, while for p-type NiO NTs, the Fermi energy level is closer to the valence band [91]. Furthermore, the conduction band edge of TiO_2_ is 0.5 eV less than that of NiO [92]. When TiO_2_ NTs and NiO NPs couple a nanoscale p-n heterostructure of TiO_2_/NiO is formed. Some electrons in the TiO_2_ NTs fill the holes in the NiO NPs semiconductor because these holes can be found in lower-energy states. This generates positively charged cores in the TiO_2_ NTs and negatively charged electrons in the NiO. The space charge layer induces an internal electrical field by polarization. The built-in electron field leads to changes in the energy band bending of the TiO_2_ NTs and NiO NPs at the interface [93].

The energy band in TiO_2_ NTs shifts downward whereas the energy band in NiO NPs shifts upward until the Fermi level reaches its new equilibrium state. The electron-hole lifespan will be extended as a result of the new Fermi level equilibrium condition. Under light illumination of sufficient energy, photons incident on TiO_2_ NTs (or NiO NPs) and generate electron/hole pairs in the electric field. Photogenerated holes flow into the negative field, whereas photogenerated electrons flow into the positive field, because of the inner electric field, i.e., the inner electric field accelerates the holes in the valence band of TiO_2_ NTs toward the p-type NiO NPs, and the electrons from NiO NPs toward n-type TiO_2_ NTs. Therefore, the built-in electric field acts as a potential barrier that prevents the photoexcited electrons-holes upon light illumination of sufficient energy. This enabled the creation of extra free carriers on the active sites of the TiO_2_/NiO nanocomposites. Furthermore, this field can be a benefit in increasing the charge mobility to the catalyst, which results in an improvement of photochemical reactivity [94].

The holes at the valence band react with H_2_O or hydroxide ions (OH^−^) to generate short-lived hydroxyl radicals (${\mathrm{OH}}^{.}$), which is the primary reactive oxidizing species. Simultaneously, the electrons at the conduction band are trapped by adsorbed oxygen molecules (O_2_) that produce highly active superoxide anion radicals ($O_{2}^{.-}$) and hydrogen peroxide (H_2_O_2_). Electrons are trapped at molecular oxygens, preventing the recombination of charge carriers and consuming electrons efficiently. These radicals ($O_{2}^{.-}$ and ${\mathrm{OH}}^{.}$) are powerful reactive species that aggressively attack organic compounds adsorbed on the surface of the photocatalyst [31]. At the same time, photogenerated electron/hole pairs can be formed by the light absorption of the dye. The electrons in the MB dye can be transferred to the conduction band (C.B) of TiO_2_/NiO and trapped by oxygen to generate free radicals [95]. The free radicals can initiate the oxidative degradation of MB pollutants down to water and carbon dioxide (H_2_O and CO_2_) [47]. However, it has been reported that the valence band hole potential is positive enough to oxidize the adsorbed organic pollutants [96]. Thus, it was suggested that two types of reaction pathways may be considered for the oxidation of organic pollutants on the TiO_2_ NTs.

The mechanism of degradation of MB dye over TiO_2_/NiO photocatalyst can be summarized in the below equations:

(10) $${\mathrm{TiO}}_{2}/\mathrm{NiO}\;+h\nu \;\to {\;\mathrm{TiO}}_{2}/\mathrm{NiO}\;\left(e^{-}{\;}_{\mathrm{CB}}+{\;h}^{+}{\;}_{\mathrm{VB}}\right)$$

(11) $$\mathrm{dye}+\;h\nu \;\to \;\mathrm{dye}\;\left(e^{-}{\;}_{\mathrm{CB}}+{\;h}^{+}{\;}_{\mathrm{VB}}\right)$$

(12) $$\mathrm{dye}\left(e^{-}{\;}_{\mathrm{CB}}+h^{+}{\;}_{\mathrm{VB}}\right)+{\mathrm{TiO}}_{2}/\mathrm{NiO}\to {\mathrm{TiO}}_{2}/\mathrm{NiO}\left(e^{-}{\;}_{\mathrm{CB}}\right)+\;\mathrm{dye}\left(h^{+}{\;}_{\mathrm{VB}}\right)$$

(13) $$h^{+}+{\;H}_{2}O\;\to {\;\mathrm{OH}}^{•}+{\;H}^{+}$$

(14) $$h^{+}+{\mathrm{OH}}^{-}\to {\;\mathrm{OH}}^{•}$$

(15) $$e^{-}+{\;O}_{2}\;\to {\;O}_{2}^{\;•-}$$

(16) $$e^{-}+{\;O}_{2}^{\;•-}+2{\;H}^{+}\;\to {\;H}_{2}O_{2}$$

(17) $$e^{-}+{\;H}_{2}O_{2}+{\;H}^{+}\;\to {\;\mathrm{OH}}^{•}+{\;H}_{2}O$$

(18) $$e^{-}+{\;H}_{2}O_{2}\;\to {\;\mathrm{OH}}^{•}+{\;\mathrm{OH}}^{-}$$

(19) $$e^{-}+{\;O}_{2}+{\;H}^{+}\to {\;\mathrm{HO}}_{2}^{\;•}$$

(20) $${\mathrm{OH}}^{•},{\;O}_{2}^{\;•-},H_{2}O_{2}+\mathrm{dye}\left(h^{+}{\;}_{\mathrm{VB}}\right)\;\to \;\mathrm{products}\;\left({\mathrm{CO}}_{2}+{\;H}_{2}O\right)$$

(21) $$h^{+}+\mathrm{dye}\;\to \;\mathrm{product}\;\left({\mathrm{CO}}_{2}+{\;H}_{2}O\right)$$

## 4. Conclusions

In summary, solar-light-driven TiO_2_/xNiO nanocomposites were successfully prepared by hydrothermal method with different NiO NPs weight ratios. The prepared samples have been described by XRD, EDX, SEM, UV-visible, and FTIR for morphological and structural characterization. The photocatalytic degradation of MB was studied and the effects of the initial dye concentration, TiO_2_/NiO dosage, and exposure time on removal efficiency were examined. After loading NiO nanoparticles with a reduced bandgap, the optical response of TiO_2_ NTs in the visible region was improved. The TiO_2_/2NiO showed better photocatalytic efficiency than the pure TiO_2_ NTs and NiO nanopowders against the MB dye. The MB photodegradation efficiency was enhanced due to the induced formation of the p-n nanocomposite in the TiO_2_/NiO that prevents electron-hole recombination. The mechanism of charge transfer under solar light irradiation inside the TiO_2_/NiO nanocomposite structure has been investigated. Our findings show that, due to its high photocatalytic capability and stability, the TiO_2_/2NiO nanocomposite shows potential for a wide range of applications as efficient photocatalysts in wastewater purification.

## Figures and Tables

**Table 1:** The structural parameters of the photocatalysts from XRD data; crystallites sizes (D), interplanar distances (d), and texture coefficients (TC).

Sample	hkl	2θ	d (A°)	D (A°)	TC
**TiO_2_ NTs**	**(201)**	24.41	3.65	72.83	0.76
	**(002)**	28.69	3.11	58.76	1.87
	**(−** **311)**	33.51	2.67	99.22	0.84
	**(311)**	39.29	2.29	50.35	0.50
	**(020)**	48.25	1.89	252.6	2.22
	**(512)**	61.77	1.5	55.26	0.56
	**(520)**	69.82	1.35	57.83	0.25
**NiO NPs**	**(111)**	37.30	2.41	352.5	1.15
	**(200)**	43.32	2.09	359.4	1.89
	**(220)**	62.91	1.48	301.6	0.72
	**(311)**	75.41	1.26	501.7	0.24
**TiO_2_/2NiO** **(NiO crystallites)**	**(111)**	37.37	2.41	352.6	1.14
	**(200)**	43.39	2.09	312.5	1.86
	**(220)**	63.01	1.48	341.2	0.75
	**(311)**	75.52	1.26	803	0.25

**Table 2:** The kinetic parameters of the studied models for MB photodegradation using different photocatalysts.

Samples	Zero		First		Second	
	K_0_	R^2^	K_1_	R^2^	K_2_	R^2^
**TiO_2_ NTs**	−0.01603	0.86242	0.02838	0.96471	0.05529	0.99367
**TiO_2_/1NiO**	−0.01540	0.76118	0.03245	0.92756	0.08020	0.93822
**TiO_2_/2NiO**	−0.01848	0.67713	0.09682	0.96600	1.19736	0.90219
**TiO_2_/3NiO**	−0.01270	0.85215	0.01865	0.93224	0.02868	0.97726
**NiO NPs**	−0.01646	0.84477	0.03087	0.96170	0.06487	0.99384

**Table 3:** Comparison of the efficiency between the present study and previously reported works of photodegradation of MB dye.

Catalyst	Light Source	Irradiation Time (min)	Dye Removal (%)	Ref.
**1TiO_2_/2NiO**	Solar Light	25	99.5	Present work
**CdS/TiO_2_**	Solar Light	30	91.9	[85]
**activated carbon/ TiO_2_**	Micro-Wave	60	99	[86]
**Cr_2_S_3_/GO/TiO_2_**	visible light	120	98.3	[87]
**Graphene/silica/TiO_2_**	visible light	60	98	[88]
**TiO_2_/zeolite/Ni**	UV irradiation	120	99.08	[89]

## Data Availability

Not applicable.

## Supplementary Materials

The following are available online at https://www.mdpi.com/article/10.3390/nano12060989/s1, Figure S1(a,b). Histograms of the particle size distribution for (a) NiO and (b) TiO_2_/2NiO; Figure S2. The photocatalytic performance of TiO_2_/2NiO composite versus the performance of TiO_2_/2NiO mixture.
