# Supplementary material for: Fabrication of TiO2/NiO p-n Nanocomposite for Enhancement Dye Photodegradation under Solar Radiation
Source: Nanomaterials (Basel). 2022 Mar 17;12(6):989. doi: 10.3390/nano12060989 (PMC8950902; doi:10.3390/nano12060989)
Supplement: Supplementary file 1 [file nanomaterials-12-00989-s001.zip › nanomaterials-1610623-supplementary.pdf]

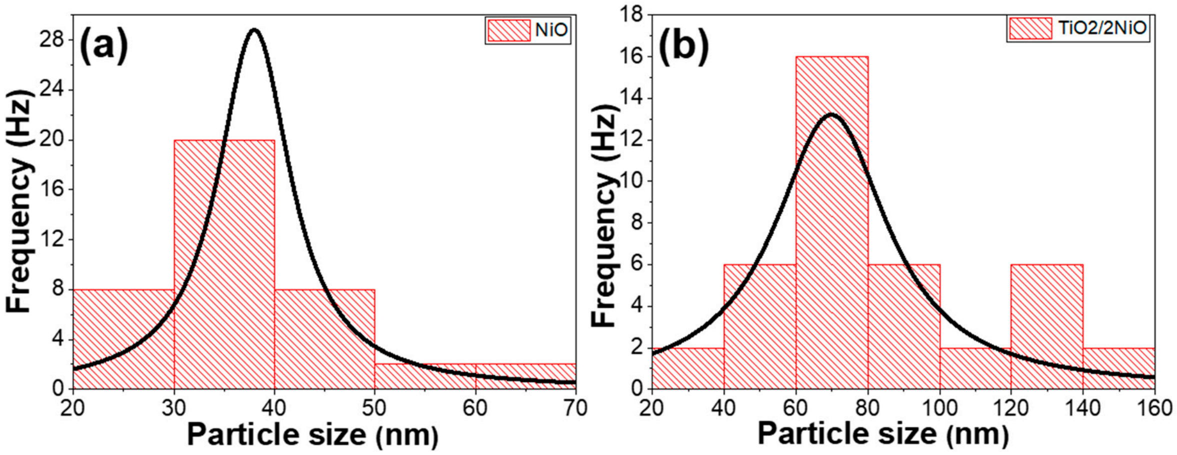

Figure S1. Histograms of the particle size distribution for (**a**) NiO and (**b**) TiO_2/2NiO.

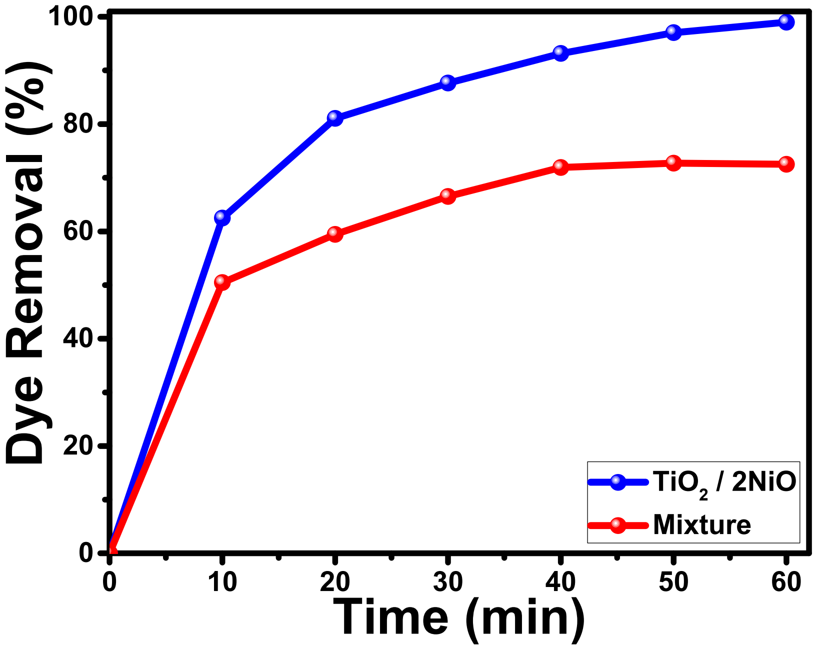

Figure S2. The photocatalytic performance of TiO_2/2NiO composite versus the performance of TiO_2/2NiO mixture.

As shown in Figure S1, we combined TiO_2 and NiO particles with ratio 1:2 and investigated the photocatalytic activity of the TiO_2/2NiO mixture versus the TiO_2/2NiO composite. The results demonstrate that the TiO_2/2NiO composite (approximately 99.8% after 60 min) has superior photocatalytic performance in comparison to the TiO_2/2NiO mixture (about 58.9% after 60 min). This is due to the TiO_2/2NiO composite generating p-n junctions.
